# Lunar Phase-Dependent Expression of Cryptochrome and a Photoperiodic Mechanism for Lunar Phase-Recognition in a Reef Fish, Goldlined Spinefoot

**DOI:** 10.1371/journal.pone.0028643

**Published:** 2011-12-07

**Authors:** Masato Fukushiro, Takahiro Takeuchi, Yuki Takeuchi, Sung-Pyo Hur, Nozomi Sugama, Akihiro Takemura, Yoko Kubo, Keiko Okano, Toshiyuki Okano

**Affiliations:** 1 Department of Electrical Engineering and Bioscience, Graduate School of Advanced Science and Engineering, Waseda University, Tokyo, Japan; 2 Department of Chemistry, Biology, and Marine Science, Faculty of Science, University of the Ryukyus, Okinawa, Japan; 3 Precursory Research for Embryonic Science and Technology, Japan Science and Technology Agency, Tokyo, Japan; Vanderbilt University, United States of America

## Abstract

Lunar cycle-associated physiology has been found in a wide variety of organisms. Recent study has revealed that mRNA levels of *Cryptochrome* (*Cry*), one of the circadian clock genes, were significantly higher on a full moon night than on a new moon night in coral, implying the involvement of a photoreception system in the lunar-synchronized spawning. To better establish the generalities surrounding such a mechanism and explore the underlying molecular mechanism, we focused on the relationship between lunar phase, *Cry* gene expression, and the spawning behavior in a lunar-synchronized spawner, the goldlined spinefoot (*Siganus guttatus*), and we identified two kinds of *Cry* genes in this animal. Their mRNA levels showed lunar cycle-dependent expression in the medial part of the brain (mesencephalon and diencephalon) peaking at the first quarter moon. Since this lunar phase coincided with the reproductive phase of the goldlined spinefoot, *Cry* gene expression was considered a state variable in the lunar phase recognition system. Based on the expression profiles of *SgCry*s together with the moonlight's pattern of timing and duration during its nightly lunar cycle, we have further speculated on a model of lunar phase recognition for reproductive control in the goldlined spinefoot, which integrates both moonlight and circadian signals in a manner similar to photoperiodic response.

## Introduction

Synchronous reproductive behaviour is a phenomenon that occurs when all individuals within a population reproduce at a specific phase set by certain periodic changes in the environment. This is an important adaptive strategy in organisms that enables higher reproductive efficiency, decreased predation risk, and the prevention of interspecies mating [1]. In addition to daily and annual cyclic changes in the terrestrial and aquatic environments, lunar cycle-dependent changes are used as cues for synchronous behavior in some species. The lunar- or semilunar-synchronized reproduction has been seen in a wide variety of living organisms in multiple phyla [2]. Although lunar- or semilunar-synchronized spawning behaviors have been demonstrated in corals [3], [4], marine insects [5], certain groupers [6], spinefoots [7], [8] and others, molecular mechanism(s) underlying this behavior remain elusive. Several factors combined have been suggested to determine the reproductive timing in corals. For example, the month of spawning is set by cycles in solar radiation and local weather pattern, the date of spawning is set by the lunar or tidal cycle, and the hour and minute of spawning is set by sunset time [9]. Responsiveness to the amount of moonlight may also be a factor as shown by the coral mRNA levels of *Cryptochrome* (*Cry*) which were significantly higher on full moon nights than on new moon nights [10]. Another well-studied organism is a marine midge *Clunio marinus* which shows (semi)lunar rhythms in reproductive behavior even in laboratory condition, and the rhythms are likely controlled by a combination of circadian clocks, moonlight, tidal fluctuations and temperature [5].

CRY proteins are highly related to (6–4) photolyases, enzymes repairing UV-induced DNA damage with photon energy [11], [12], and contain FAD chromophore [13], [14]. The photosensory properties of cryptochromes (CRYs) have been investigated in a plant *Arabidopsis thaliana* and a fruit fly *Drosophila melanogaster*, and they are shown to play important roles in photomorphogenesis and circadian photoreception, respectively [15], [16], [17]. In *Drosophila* circadian photoreception, illumination triggers interaction between dCRY and dTIMLESS [18], [19], both of which are important factors for circadian clock oscillation and resetting. This interaction results in the degradation of both factors, and consequently another critical factor dPER (Period) becomes destabilized [20]. These events are crucial for phase-shift of the *Drosophila* circadian clock. In vertebrates, CRY1 and CRY2 are considered to lack the photosensitivity but instead constitute negative feedback loops in the circadian clock system together with PERIOD, CLOCK, and BMAL [21], [22]. The moonlight-associated upregulation of *Cry* mRNA in coral [10] does not necessarily establish coral CRY as a moonlight-sensitive photoreceptor, but it does imply the involvement of CRY in lunar-synchronized spawning. Because the corals spawn during a full moon, it is difficult to infer whether *Cry* mRNA levels are directly associated with a putative “circalunar clock” that contains oscillatory factors called state variables determining the phase of the oscillation or whether they are responsive only to the intensity and/or the duration of moonlight. In terms of other lunar-responding animals, there have yet to be any molecular observations of a potential gene involved in the lunar-dependent spawning.

Investigation of the relationship between lunar phase, *Cry* gene expression patterns, and the lunar-dependent spawning response in animal species other than corals could provide better understanding of a lunar phase-recognition mechanism as well as the generalities surrounding a lunar-associated change of *Cry* gene expression across animal species. In this study, we focused on the *Cry* genes of a lunar-synchronized spawner, the goldlined spinefoot (formerly referred to as the golden rabbitfish or orange-spotted spinefoot) (*Siganus guttatus*). The goldlined spinefoot is a reef fish with a lunar-dependent rhythmic spawning restricted to a species-specific lunar phase (around the first quarter moon) during spawning season [7], [8], [23], [24], [25]. Two *Cry* genes, *SgCry1* and *SgCry3*, were identified as showing lunar cycle-dependent expression that peaked at the first quarter moon in the goldlined spinefoot brain, leading us to a concept that *SgCry* genes do not simply respond to the intensity of moonlight, but they are state variables in a lunar phase-recognition system.

## Results

### Cloning and phylogenetic analysis of *SgCry1* and *SgCry3*

**Figure 1:**
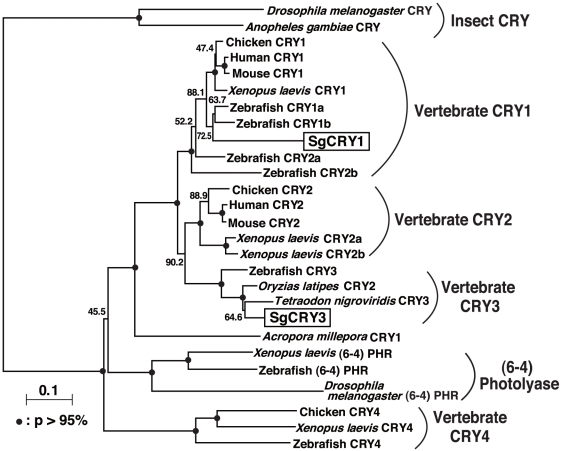
Phylogenetic tree of CRY family proteins. Goldlined spinefoot CRY sequences (SgCRY1 and SgCRY3; present study) and their related sequences obtained from NCBI Entrez database (accession nos. are shown in Table S1) were analyzed using the Neighbor-Joining method [44] and CLUSTAL W (http://clustalw.ddbj.nig.ac.jp/top-e.html). Bootstrap probabilities (p) are represented by closed circles on the nodes (p>95%) or values near the nodes.

We identified two kinds of *Cry* genes in the juvenile goldlined spinefoot. Phylogenetic analysis (Figure 1) showed that they can be classified in vertebrate CRY1 and CRY3 groups, respectively, which led us to identify them as SgCRY1 (AB643455) and SgCRY3 (AB643456). In the Nighbor-Joining phylogenetic tree (Figure 1), *Cry3* genes seem to form a monophyletic group with *Cry2* genes, which strongly suggests that *Cry3* genes are fish orthologs of *Cry2* genes.

### Weekly changes in female GSI

**Figure 2:**
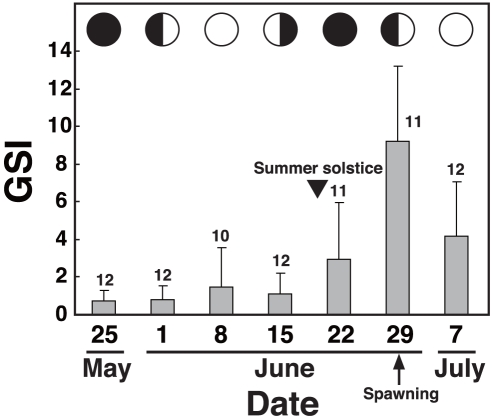
Temporal change in GSI values of the female goldlined spinefoot during the lunar reproductive cycle. Gonadosomatic index (GSI) was calculated as described in Materials and Methods. Numbers of fish sampled are indicated above the bars. An arrow and an arrowhead show the spawning and the summer solstice, respectively. Indicated by seven schematic images are lunar phases from new moon (May 25^th^, left) to full moon (July 7^th^, right).

In order to evaluate the mRNA expression profiles of *SgCry* mRNA expression during sexual maturation of the female goldlined spinefoot, we focused on medial part of the brain (mesencephalon and diencephalon) and the ovary since these sites are closely associated with reproduction in vertebrates. We collected the medial part of the brain and ovary from mature fish for approximately 2 months at 7- or 8-day intervals during and just before the first spawning. Concomitantly, we measured the gonadosomatic index (GSI, Figure 2) to confirm that the fish had sexually matured during the study period. GSI did not significantly change from May 25^th^ to June 15^th^, 2009, but did increase to a peak on June 29^th^, 2009 (first quarter moon) and significantly decreased (P < 0.01) on July 7^th^, 2009 (full moon), indicating that June 29^th^, 2009 was the first of the two spawning phases during this reproductive season.

### Daily variations of *SgCry1* and *SgCry3* mRNA expression

**Figure 3:**
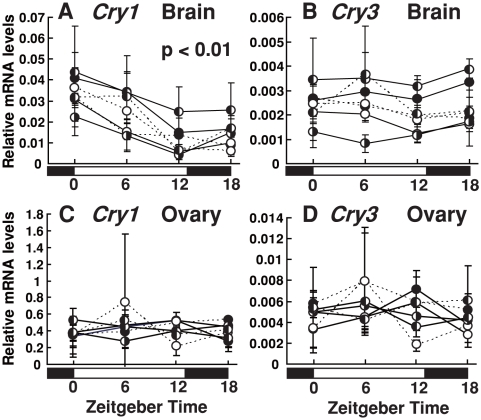
*SgCry* mRNA levels and their daily variation in the brain and ovary. Each tissue (brain [mesencephalon and diencephalon]: n = 4, ovary: n = 3) was collected at ZT0, ZT6, ZT12, ZT18 from new moon to full moon over one and half lunar cycle. Each *Cry* mRNA level was calculated as a value relative to that of the *Sg*β*-actin* gene. Error bars represent ± SD. Lunar phases are indicated by schematic moon images. Solid lines show the first lunar cycle, and broken lines show second lunar cycle. (A) Daily changes in the *SgCry1* mRNA levels in the brain. p<0.01, Two-way ANOVA. (B) Daily changes in the *SgCry3* mRNA levels in the brain. (C, D) Daily changes in the *SgCry1* and *SgCry3* mRNA levels in the ovary. The graphs are separated into single panels for each lunar phase in Figure S1.

We investigated daily variations in *SgCry1* and *SgCry3* mRNA expression and lunar phase-dependent change in the expression pattern using quantitative RT-PCR and cDNA synthesized from the brain and ovary RNA (Figure 3, and Figure S1). Abundances of *SgCry1* mRNA in the brain fluctuated with peaks at dawn (ZT0 [Zeitgeber time 0]) and gradual decreases during the daytime (Figure 3A). Although the averaged *SgCry1* mRNA expression levels diverged depending on lunar phase (see below), the expression patterns were similar (Figure 3A). On the other hand, mRNA levels of *SgCry3* were almost constant in the brain throughout the day and during all lunar phases (Figure 3B). In the ovary, daily variation was not observed for *SgCry1* and *SgCry3* mRNA expression (Figure 3C, D) except that weak daily variations were observed for *SgCry1* and *SgCry3* mRNA expression at June 1^st^ and May25^th^, respectively (p<0.05, Figure S1).

### Lunar variations of *SgCry1* and *SgCry3* mRNA expression

**Figure 4:**
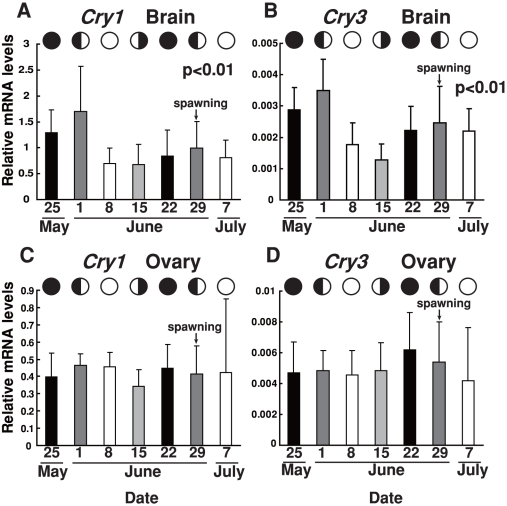
Lunar phase-dependency of *SgCry* mRNA expression in the brain and ovary. Each *Cry* mRNA was calculated as a value relative to that of the *Sg*β*-actin* gene. For each lunar phase, mRNA levels at ZT0, ZT6, ZT12 and ZT18 are averaged except for *SgCry1* mRNA levels in the brain (mesencephalon and diencephalon). Since *SgCry1* showed diurnal variation in the brain, values were normalized to reduce the deviation due to the daily rhythmic changes as follows; values at each daily time point (ZT0, ZT6, ZT12, and ZT18) were divided by the mean values of all the data for the same time point (0.0340, 0.0242, 0.0109, and 0.0141 for ZT0, ZT6, ZT12, and ZT18, respectively). Error bars represent ± SD. Lunar phases are indicated by schematic moon images. (A) Lunar changes in the *SgCry1* mRNA levels in the brain. p<0.01, One-way ANOVA. Post hoc test; p<0.05, 5/25 vs 6/8, 6/15; p<0.01, 6/1 vs 6/8, 6/15, 6/22, 6/29, 7/7). (B) Lunar changes in the *SgCry3* mRNA levels in the brain. p<0.01, One-way ANOVA. Post hoc test; p<0.05, 6/15 vs 6/22, 7/7; p<0.01, 5/25 vs 6/8, 6/15; p<0.01, 6/1 vs 6/8, 6/15, 6/22, 6/29, 7/7; p<0.01, 6/15 vs 6/29. (C, D) *SgCry* mRNA levels in the ovary plotted against lunar phase.

We reanalyzed the results of quantitative RT-PCR from the perspective of possible lunar phase-dependent variation in *SgCry1* and *SgCry3* mRNA levels (Figure 4). Both *SgCry1* and *SgCry3* mRNA expression in the brain showed significant lunar-dependency (p<0.01; Figure 4A, B). Expression was highest on June 1^st^ (first quarter moon), showed decrease on June 8^th^ (full moon), and the lowest expression around June 8^th^ and June 15^th^ (last quarter moon). There were also fluctuations in both expression levels with peaks on June 29^th^ (first quarter moon). In contrast to the clear lunar phase-dependency in the brain, levels in the ovary did not show much significant variation (Figure 4C, D).

## Discussion

In this study, we identified two cryptochrome genes in the goldlined spinefoot, *SgCry1* and *SgCry3*, and investigated their mRNA levels in the brain (mesencephalon and diencephalon) and ovary over the two months comprising the reproductive season in this fish species. *SgCry1* mRNA levels showed diurnal variation with a peak at dawn (ZT0, Figure 3A), indicating that *SgCry1* mRNA expression was under circadian clock control and/or environmental light signals. In contrast to the brain, *SgCry1* mRNA levels in the ovary did not show strong diurnal fluctuation (Figure 3C, and Figure S1). This finding is analogous to our previous observation that mRNA levels of *Xenopus tropicalis Cry1* and *Cry2* did not significantly differ between midday and midnight in the ovary [26]. CRYs in the ovary might be involved in physiological mechanisms other than circadian function.

Reanalysis of *SgCry* mRNA expression in relation to lunar variation revealed that the expression of both *Crys* has strong lunar phase-dependency in the brain but not in the ovary (Figure 4A, B). *SgCry1* mRNA expression demonstrated diurnal and lunar variation, while *SgCry3* mRNA expression demonstrated only lunar phase-dependent variation. These *SgCry* mRNA variations were highest at the first quarter moon and then decreased before the full moon. Such rhythmic changes were observed in not only the lunar cycle in relation to spawning (June 29^th^) but also the preceding cycle (May 25^th^ – June 15^th^), indicating that these changes are not simply associated with spawning. Because we reared the fish in this study under natural photoperiod and moonlight without exposure to the tidal cycle, which has an approximately two-week periodicity, it is highly probable that the environmental cue leading to the perception of lunar phase is not the tide but rather moonlight. In fact, some authors have previously revealed that the goldlined spinefoot is responsive to full moonlight such that there are affects on nocturnal melatonin [27] and *Period2* mRNA levels in the pineal gland [28]. In addition to retinal photoreceptors, pineal or encephalic photoreceptors such as exorhodopsin [29], pinopsin [30], parapinopsin [31] and encephalopsin [32] may exist in the fish, and they may be coordinately involved in the system for discriminating strong solar light (∼1×10^5^ Lux) and weak moonlight (less than 1 Lux).

SgCRY1 and SgCRY2 are classified into vertebrate CRY1 and CRY2 groups, respectively (Figure 1). CRYs in the CRY1 and CRY2 groups are called “mammalian type” CRYs [33] and considered to be non-photoreceptive [34] in contrast to photoreceptive “insect type” CRYs (insect CRYs, Figure 1). However, recent studies suggest the photoreceptive nature of the mammalian-type CRYs [35], [36], and therefore SgCRYs might also be candidate photoreceptors for moonlight perception in the goldlined spinefoot, as has been suggested for coral [10]. This idea supports the present result that *SgCry3* mRNA levels were highest at the first quarter moon.

Although a mechanism for the detection of moon phase has yet to be identified, the present results imply a general mechanism relevant to a lunar-associated change of *Cry* gene expression across animal species. The simplest possible mechanism for the detection of moon phase would utilize maximal moonlight intensity and/or the length of a light period that exceeds a certain light intensity. Such a mechanism, however, would be useful for detecting the full moonlight, as in the case of corals, but may be inappropriate for detecting the quarter moons. Therefore, alternative mechanism is likely used at least in the goldlined spinefoot. Another possible mechanism that could explain the recognition of specific lunar phase is a mechanism similar to that for photoperiodic reproductive control, which involves the circadian clock and photoreception. In fact, photoperiodic influences of the moon on the regulation of (semi)lunar-rhythm has been postulated and experimentally explored in a marine midge *C. marinus* [5].

**Figure 5:**
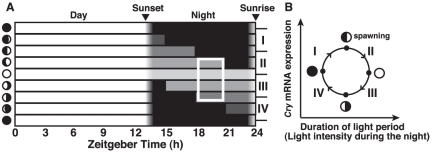
Models for photoperiodic regulation of *SgCry* expression by moonlight and for a lunar limit cycle. (A) Schematic of solar light phase in Okinawa, moonlight phase, and moonlight intensity during the experiments. A putative photo-repressive phase is indicated by white frame. (B) Lunar limit cycle model served by *SgCry* as a state-variable. *SgCry* mRNA level decreases from first lunar quarter to last lunar quarter when moonlight intensity is relatively high and exposed time of moonlight is relatively long (Phase II, Phase III. *SgCry* mRNA levels increase from last lunar quarter to first lunar quarter (Phase IV–Phase I).

In birds, there are photoreceptors in the deep brain that detect environmental light information [37], [38], which is then transmitted to the mediobasal hypothalamus (MBH), followed by the regulation of gonadal maturation. In the MBH of long-day responsive quails, day length is measured according to the light conditions surrounding a specific phase in the early night called the “photo-inducible phase” [39], [40]. Previous studies have shown that photoperiodism also induces gonadal maturation in fish such as masu salmon (*Oncorhynchus masou*, [41]), rainbow trout (*O. mykiss*, [42]), and gilthead seabream (*Sparus aurata*, [43]). Moonlight is seen to periodically change both in intensity and duration (timing of moonrise and moonset) in accordance with the moon phase (Figure 5A). When comparing the moonlight patterns (Figure 5A) to the lunar-dependent change in *SgCry* mRNA expression (Figure 4A, B), we noted a decrease in *SgCry* mRNA levels from the first quarter moon to full moon that paralleled the increase in light intensity during ZT18–ZT21. Therefore, we are speculating that the mRNA expression of *SgCry* is controlled by the light conditions during ZT18–ZT21. That is, *SgCry* mRNA expression is repressed in moon phases with moonlight irradiation during ZT18–ZT21 (phase II and III, Figure 5A, B) and activated in moon phases without moonlight irradiation during ZT18–ZT21 (phase IV and I, Figure 5A, B). If so, the lunar phase-dependent gene expression could be regulated by a moonlight signal given during the “photo-repressive phase”, a specific circadian time similar to the photo-inducible phase in photoperiodism.

The lunar phase-dependent expression of *SgCry*s, which has a cycle advanced by one fourth that of the lunar cycle, raises the possibility that *SgCry* could function as a state variable determining the lunar phase. In this system, *SgCry* expression and moonlight intensity (or another state variable reflecting the moonlight intensity) would form a hypothetical lunar limit cycle (Figure 5B), and the combination of these two factors might constitute a putative “circalunar clock” and enable the goldlined spinefoot to recognize any specific lunar phase as a point within this limit cycle.

In consideration of the expression patterns, *SgCry* genes are likely under a combined regulation of circadian clock- and moonlight-responsive element(s), or alternatively they are regulated by a novel cis-element, the activity of which changes with the peak seen around the first quarter moon (a lunar phase-responsive element). Further analyses of the transcriptional regulatory mechanisms of *SgCry1* and *SgCry3* genes in the brain would shed light on the precise mechanism(s) for the lunar phase-recognition system that serves the oocyte maturation processes and decides the timing of spawning through induced synthesis or secretion of GnRH in the brain.

## Materials and Methods

### Experimental Fish

Experimental animal care was conducted under permission from the Committee for Animal Experimentation of the School of Science and Engineering at Waseda University (permission # 09A03). Animal experiments including sampling in the field were conducted under permission from Sesoko Station Tropical Biosphere Research Center at University of the Ryukyus (permission # 090522–090707). Juvenile goldlined spinefoot fish (0.08–0.15 g) were originally collected by using a fish net at low tide around the new moon period from a mangrove swamp of the Manna River in Okinawa, Japan. The fish were reared in outdoor tanks (capacity: 10 metric tons) with aerated running seawater for three or four years under natural photoperiod, natural moonlight, and water temperature conditions at Sesoko Station, Tropical Biosphere Research Center, University of the Ryukyus, Japan. There are roofs and frames over the tanks to protect them from rain and wind, but the sunlight and moonlight reach to the tanks. There is no artificial light possibly reaching the fish inside the tanks. The feeding of commercial pellets (EP1, Marubeni Nisshin, Tokyo, Japan) took place daily at 10:00 h. Approximately 3-year-old or 4-year-old fish with body weights ranging from 200 to 700 g were collected by using a fish net weekly from May 25^th^ to July 7^th^, 2009 and used in the present experiments. During the experiment, sunrise and sunset occurred at approx. 6:00 and 19:00, respectively. The fish were taken from the tanks at random and anesthetized with iced seawater in a bucket. The fish were brought to the laboratory within a few minutes under natural light condition (in daytime sampling [06:00–18:00]) or with covering the bucket to keep the fish in the dark (in nighttime sampling [24:00]). The brain (medial part of the brain, mesencephalon and diencephalon) (n = 4) and ovary (n = 3) were collected from the fish at 06:00 (∼ZT0 [Zeitgeber time 0]), 12:00 (∼ZT6), and 18:00 (∼ZT12) under fluorescent light or at 24:00 (∼ZT18) under dim red light (<0.1 mW/cm^2^,>640 nm). In the sampling at 24:00, the eyes of each fish were covered with aluminum foil during decapitation to minimize an effect of dim red light. Samples were kept in RNA*later* (Ambion) at 4°C overnight and then stored at −80°C until RNA extraction. During the sampling, body mass and ovarian mass were recorded separately. Gonadosomatic index (GSI) was calculated using the following formula: GSI = (ovarian mass/body mass)×100.

### Complementary DNA Cloning of *SgCry1* and *SgCry3* Genes Encoding Full-Length Coding Sequences

Total RNA was extracted from juvenile goldlined spinefoot using TRIzol reagent (Invitrogen). First-strand cDNAs were synthesized with SuperScript III reverse transcriptase (Invitrogen) using KSII(dT)_21_ primer (GAGGTCGACGGTATCGATAAGC(T)_21_). The *SgCry1* and *SgCry3* cDNA fragments were amplified using a pair of degenerate primers (5′-GTCCTSGAYCCBTGGTTYG-3′ and 5′-GTCATDATNGCRTCDATCC-3′) that were designed based on the conserved regions of *Cry* genes from some species (*Acropora millepora*, *Gasterosteus aculeatus*, *Oryzias latipes*, *Takifugu rubripes*, *Xenopus tropicalis*, and *Gallus gallus*). Complementary DNAs including the 5′ and 3′ UTRs were obtained using RACE. cDNAs that included the entire coding sequence for *SgCry1* and *SgCry3* were obtained by PCR with primers for their 5′ and 3′ UTRs using *PfuUltra* (Stratagene). The amplified fragments were inserted into the pENTR/D-TOPO vector (Invitrogen), and the inserts of at least three independent clones for each gene were sequenced. Finally, *SgCry1* and *SgCry3* cDNAs for the CDS without presumed PCR errors were isolated.

### Quantitative RT-PCR Analysis

Total RNA was extracted from the tissues using TRIzol reagent (Invitrogen). Residual genomic DNA in the total RNA sample was eliminated by DNase I treatment (RNase-free recombinant DNase I, TaKaRa BIO). Quantitative RT-PCR analyses were performed using StepOnePlus (Applied Biosystems) along with a high capacity cDNA reverse transcription kit (Applied Biosystems). Each reaction included 1 µg of total RNA as a template. The primers for quantitative RT-PCR are shown in Table 1. In order to establish a consistently transcribed gene for reference, we isolated and sequenced partial cDNA from *SgrpL13A* (AB643457), *SgPGK* (AB643458), *SgEF1*α (AB643459), *Sg*β*-actin* (AB643460), and *Sg*β*2M* (AB643461) of the juvenile fish cDNA. We examined the performance of one or more pairs of the primer for each gene by amplification of the cDNA in the presence or absence of RTase, electrophoresis of the amplified fragments. Because we did not obtain primer sets giving specific amplification and a single band for two of the five genes (*SgrpL13A* and *Sg*β*2M*), the other three genes with sufficient performance (*SgPGK*, *SgEF1*α, *Sg*β*-actin*) were used in the further analysis. Among these genes, we selected *Sg*β*-actin* as a reference control gene in the following measurements since the threshold cycles (Ct) for *Sg*β*-actin* were relatively unchanged over the sampling time (Figure S2).

**Table 1:** Primers for quantitative RT-PCR.

primer	Sequence	length	%GC	Tm
Sg Cry1 RT_PCR_F	TAGAGGATTTGGACGCCAGCCTAC	24	54.1	62.1
Sg Cry1 RT_PCR_R	CAGCCTCACTGGCTAGTTTATGGAC	25	52.0	62.1
Sg Cry3 RT_PCR_F	GGTGTGGAGACTATTGTCAGAAACTCA	27	44.4	60.5
Sg Cry3 RT_PCR_R	CTTCCAGCGATGGGATACTGTATAAC	26	46.1	60.4
Sg_pgk_qRT-PCR_F	CCTCAAAGTGCTCAACAACATGGAG	25	48.0	60.4
Sg_pgk_qRT-PCR_R	CTCATCGAACTTGTCAGCGGTG	22	54.5	60.4
Sg_EF1a_qRT-PCR_F	CACAGGGACTTCATCAAGAACATGATC	27	44.4	60.5
Sg_EF1a_qRT-PCR_R	CGTTCTTGGAGATACCAGCCTC	22	54.5	60.4
Sg_actin_qRT-PCR_F	CATCGCTGACAGGATGCAGAAG	22	54.5	60.4
Sg_actin_qRT-PCR_R	CTCCGATCCAGACAGAGTATTTACG	25	48.0	60.4

### Statistical Analysis

Data were analyzed using ANOVA with Tukey-Kramer multiple comparisons on Statcel2 (the add-in forms on Excel (Microsoft)) software.

## Supporting Information

Figure S1 ***SgCry*** **mRNA levels and their daily variation in the brain and ovary.**Graphs shown in Fugure 3 were separated into single panels for each lunar phase. A hash mark denotes a data set in which one of the triplicate samples was missing due to a mistake in the experiment.
(EPS)

Figure S2 **Threshold cylcles for control genes in quantitative RT-PCR analysis.**Distribution of threshold cycles for three control genes (*SgPGK*, *SgEF1*α, *Sg*β*-actin*) in the quantitative RT-PCR analysis (shown in Figure 3 and Figure 4) were shown by box plots. Outliers were omitted. Based on these plots and standard deviations of threshold cycles for *SgPGK* (brain 0.55; ovary, 1.23), *SgEF1*α (brain 0.46; ovary, 1.58), and *Sg*β*-actin* (brain, 0.41; ovary, 1.32), we selected *Sg*β*-actin* as a reference control gene.
(EPS)

Table S1 **Accession nos. of amino acid sequences used for phylogenetic analysis.**
(DOC)
