# Supplementary material for: Lunar Phase-Dependent Expression of Cryptochrome and a Photoperiodic Mechanism for Lunar Phase-Recognition in a Reef Fish, Goldlined Spinefoot
Source: PLoS One. 2011 Dec 7;6(12):e28643. doi: 10.1371/journal.pone.0028643 (PMC3233589; doi:10.1371/journal.pone.0028643)
Supplement: Table S1 — Accession nos. of amino acid sequences used for phylogenetic analysis. (DOC) [file pone.0028643.s003.doc]

| Protein | Accession No. |  |  |
| --- | --- | --- | --- |
| *Anopheles gambiae* CRY | XP_321104 |  |
| *Drosophila melanogaster* CRY | NP_732407 |  |  |
| Human CRY1 | NP_004066 |  |  |
| Mouse CRY1 | NP_031797 |  |  |
| Chicken CRY1 | NP_989576 |  |  |
| *Xenopus laevis* CRY1 | AAK94665 |  |  |
| Zebrafish CRY1a | NP_571864 |  |  |
| Zebrafish CRY1b | NP_571865 |  |  |
| Zebrafish CRY2a | NP_571866 |  |  |
| Zebrafish CRY2b | NP_571867 |  |  |
| Human CRY2 | NP_066940 |  |  |
| Mouse CRY2 | NP_034093 |  |  |
| Zebrafish CRY4 | NP_571862 |  |  |
| Chicken CRY2 | AAK61386 |  |  |
| *Xenopus laevis* CRY2a | NP001082139 |  |  |
| *Xenopus laevis* CRY2b | NP_001083936 |  |  |
| *Tetraodon nigroviridis* CRY3 | ENSTNIP00000007510 |  |  |
| *Oryzias latipes* CRY2 | ENSORLP00000018431 |  |  |
| Zebrafish CRY3 | NP_571861 |  |  |
| *Acropora millepora* CRY1 | ABP97098.1 |  |  |
| *Xenopus laevis* (6-4)PHR | NP_001081421 |  |  |
| Zebrafish（6-4）PHR | NP_571863 |  |  |
| *Drosophila melanogaster*（6-4）PHR | BAA12067 |  |  |
| Chicken CRY4 | AAQ73619 |  |  |
| *Xenopus laevis* CRY4 | NP_001088990 |  |  |
